# Multivariate-Statistical Assessment of Heavy Metals for Agricultural Soils in Northern China

**DOI:** 10.1155/2014/517020

**Published:** 2014-04-16

**Authors:** Pingguo Yang, Miao Yang, Renzhao Mao, Hongbo Shao

**Affiliations:** ^1^Shanxi Normal University, Linfen 041000, China; ^2^Shanxi Agricultural University, Taigu 030801, China; ^3^Center for Agricultural Resources Research, Institute of Genetics and Developmental Biology, Chinese Academy of Sciences, Shijiazhuang 050021, China; ^4^Key Laboratory of Coastal Biology & Bioresources Utilization, Yantai Institute of Coastal Zone Research, Chinese Academy of Sciences (CAS), Yantai 264003, China; ^5^Institute for Life Sciences, Qingdao University of Science & Technology (QUST), Qingdao 266042, China

## Abstract

The study evaluated eight heavy metals content and soil pollution from agricultural soils in northern China. Multivariate and geostatistical analysis approaches were used to determine the anthropogenic and natural contribution of soil heavy metal concentrations. Single pollution index and integrated pollution index could be used to evaluate soil heavy metal risk. The results show that the first factor explains 27.3% of the eight soil heavy metals with strong positive loadings on Cu, Zn, and Cd, which indicates that Cu, Zn, and Cd are associated with and controlled by anthropic activities. The average value of heavy metal is lower than the second grade standard values of soil environmental quality standards in China. Single pollution index is lower than 1, and the Nemerow integrated pollution index is 0.305, which means that study area has not been polluted. The semivariograms of soil heavy metal single pollution index fitted spherical and exponential models. The variable ratio of single pollution index showed moderately spatial dependence. Heavy metal contents showed relative safety in the study area.

## 1. Introduction

Soils are critical environments where rock, biology, air, and water interface. Soil pollution has become an important environmental issue in China owing to rapid economic development and industrialization and increasing reliance on agrochemicals in the last few decades [1–3]. Soil heavy metal contents are not only the serious environmental issue but also frequency related to agricultural soil utilization problem [4–6]. Soil heavy metals could be necessary or beneficial to plants at certain levels but toxic when exceeding specific threshold [7–11]. If these elements are absorbed by the plants through the root system, they may enter the food chain and become toxic to humans and animals. The ecological importance of soil heavy metals is closely related to human health due to their high ecological transference potential.

For agricultural soils, the main pollution sources of heavy metals are due to activities such as irrigation using wastewater, pesticides, agricultural fertilizers, and organic manure, disposal of urban and industrial wastes, and atmospheric pollution from motor vehicles and the combustion of fossil fuels. Heavy metals in agricultural soil have become higher than background levels. It has also become a hot spot for the study of the international soil environment science research and ensured its sustainability.

Multivariate statistical approaches including principal component analysis (PCA) and cluster analysis (CA) are the statistical tools used in the elaboration pollution [12, 13]. It has been reported that PCA methods have been widely used in geochemical applications to identify soil pollution sources and distinguish natural versus anthropic contribution [14, 15]. CA is often used coupled to PCA to check results and group individual parameters and variables [16–20]. The methods of geostatistics use the stochastic theory of spatial correlation for both interpolation and for apportioning uncertainty [21]. Geostatistics is based on the theory of a regionalized variable which uses the technique of semivariogram to measure the spatial variability of a regionalized variable and provides the input parameters for the spatial interpolation of kriging [22, 23]. Geostatistics can also be used to assess the risk of exceeding critical values (regulatory thresholds, soil quality criterion) at unsampled locations and to simulate the spatial distribution of attribute values [21].

The present study is focused on the suburban area of Shijiazhuang city. Zhengding has formed an industrial structure consisting of Shijiazhuang airport, electronic, petrochemical, and highly traffic density undergone a rapid transition from a traditionally agricultural-based to an increasingly industrial-based economy in the last 30 years which could enhance the risk of metal contamination through food chain in the region as heavy metals may enter and accumulate in agricultural soils through atmospheric deposition and irrigation. Early studies in the study area indicated the spatial variability of heavy metal distribution [17].

The study was to analyze possible sources of these heavy metals by multivariate statistical techniques and to assess soil heavy metal contamination using single pollution index and integrated pollution index to identify spatial distribution of single pollution index of soil heavy metal through geostatistical analysis.

## 2. Materials and Methods

### 2.1. Study Region and Soil Sampling

**Figure 1:**
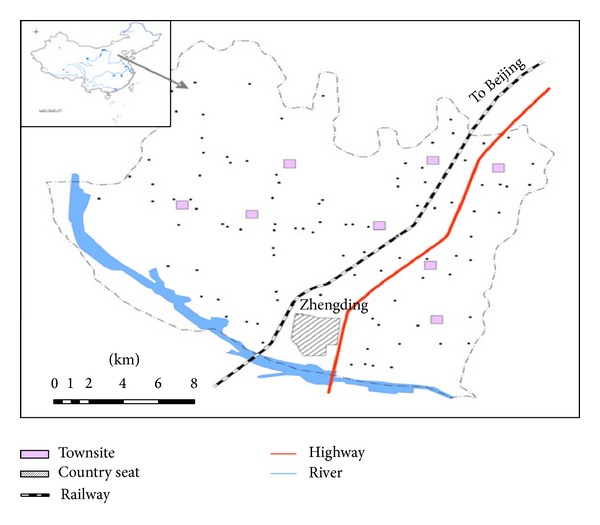
Map of the studied area and sampling locations.

The study is focused on the suburban area of Shijiazhuang city, northern China. The region is well known for intense industrial and commercial activities. Soil samples were collected from 100 locations in the Zhengding, an administrative district covering 468 km^2^ with a population of around 467,000 people. This area has a continental monsoon climate with an average annual temperature 13°C, and an average precipitation of 530 mm. The altitude levels range from 65 m to 105 m within the study area. The main soil type is carbonate cinnamon and Chao soil according to China soil classification system. The samples were collected from agricultural areas (mostly wheat, cereals, and vegetables). The coordinates of sampling locations were recorded with GPS. The sampling locations are shown in Figure 1.

### 2.2. Soil Analyses

Each sample consists of 10 soil cores (0–20 cm) depth, 1 kg ca total weight, which was collected from within a 10 × 10 m area with the central point corresponding to the defined position for the sample. All of the samples were air-dried at room temperature and sieved by a 2 mm mesh and stored in polyethene plastic bags for subsequent sample analysis. Metal concentrations for Cu, Zn, Ni, Pb, Cr, Hg, As, and Cd were analyzed after complete dissolution using a mixture of HNO_3_-HF-HCLO_4_ and heated in a microwave digestion system, using appropriate atomic absorption spectrometric techniques [17]. The accuracy of the procedure was determined by analyzing the certified reference material GB 7475-87 (National Institute of Standards and Technology, China). Quality controls involved analysis of random samples, blank samples, and national standard samples each time.

### 2.3. Assessment of Soil Contamination

The assessment of soil heavy metal contamination in agro-ecosystem is often the choice of single pollution index (Pi) and the Nemerow integrated pollution index (*P*). Generally *P*_*i*_ is defined as the ratio of the heavy metal concentration *C*_*i*_ to the second grade standard values according to the Chinese soil environmental quality standards *S*_*i*_ (GB15618-1995) [24]:

(1) $$\begin{array}{c} P_{i}=\frac{C_{i}}{S_{i}}. \end{array}$$

When *P*_*i*_ < 1, there is no metal pollution; otherwise, if *P*_*i*_ ≥ 1, it means pollution happens.

The Nemerow integrated pollution index *P* considers not only the mean values of all considered metals but also the maximum value of *P*_*i*_ [25]. *P* is determined by the mean value *P*_ave_ and the maximum *P*_max⁡_ as the following equation:

(2) $$\begin{array}{c} P=\sqrt{\frac{{P_{\max}}^{2}+{{P_{\text{ave}}}^{2}}_{}}{2^{}}}. \end{array}$$

### 2.4. Statistical Analysis

Multivariate statistical including principal component analysis (PCA), cluster analysis (CA), and geostatistical analysis are powerful tools for distinguishing pollution sources. Semivariograms model for each single pollution index considered elements (Cu, Zn, Ni, Pb, Cr, and Hg) using geostatistics. Classic statistical analyses were processed using SPSS19.0 software. Geostatistical modeling software Variowin 2.2 was used for the calculation of semivariogram of soil heavy metal single pollution index.

## 3. Results and Discussion

### 3.1. Principal Component Analysis

Summary statistics and normal test of Zhengding datasets were performed and the results were present [17]. PCA can be used to reduce data and to extract a smaller number of independent factors (principal components) to find the relationship among observed variables [16–18]. Principal component analysis (PCA) was applied in the study to have high quality experimental results. The PCA based results for soil heavy metals are listed in Table 1. According to the initial eigenvalues, three principal components are selected, accounting for over 67.8% of the total variance. The eigenvalues of the three first extracted factors are greater than one. All of the elements are consequently well represented by these three principal components.

The initial component matrix for heavy metals indicates that Cu, Zn, and Cd are associated, showing high values in the first principal component (PC1) which explains 27.3% of the total variance and loads heavily on Cu (0.65), Zn (0.88), and Cd (0.77). Cu and Zn values are controlled by a long-term anthropic activity such as pesticides. The second principal component (PC2) includes univocally Pb and As, which accounts for 20.5% of the total variance. Common sources of lead in soils are manure, sewage sludge, lead-arsenate pesticides, vehicle exhausts, and industrial fumes. The study area has a high vehicular traffic density, constituted by Zhengding international airport and several important railways, expressway (http://www.en.wikipedia.org/wiki/zhengding), for leaded gasoline widely used. The third principal component (PC3) is correlated very strongly with Hg loading (−0.79) and also by Ni loading (0.79), accounting for 20% of the total variance.

Long-term and extensive use of pesticides in farmland may cause heavy metals such as copper, nickel, zinc, and cadmium to accumulate in the topsoil [26, 27]. Common sources of lead in soils include car exhausts, manure, sewage sludge, and coal burning. Cd is present in fossil fuel such as coal and oil and having no functions in plants or animals or human body. In addition, normal agricultural practices may cause enrichment of heavy metals. These practices are an important source of Zn, Cu, and Cd due to the application of manure or inorganic fertilizers. Thus in Table 1, the relationship among all of the eight variables is clearly revealed.

### 3.2. Cluster Analysis

**Figure 2:**
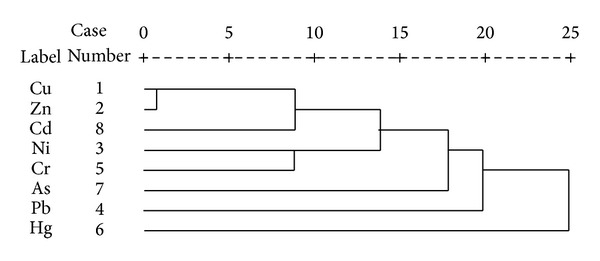
Dendrogram results from Pearson correlation coefficients of hierarchical cluster analysis for heavy metals.

Although not substantially different from PCA, CA could be used as an alternative method to confirm results and provide grouping of variables [5]. The heavy metals concentration data were calculated using the hierarchical clustering with SPSS software.  Figure 2 shows the CA results for the heavy metals as a dendrogram in the study area. This figure shows three clusters. (1) Cu, Zn, and Cd are very well correlated with each other. The farming area has had several decades of intensive tillage, long-term fertilizer and pesticide application might be a major source of accumulated heavy metals in the study area, (2) which is associated with Pb and As. Pb is mostly found in automobile battery in sufficient amount; excessive intake Pb can damage the nervous, skeletal, circulatory, enzymatic, endocrine, and immune systems of human body. (3) Ni and Hg are commonly associated in a number of rock types or soil parent materials. The analyzed results are in good accordance with the findings of the PCA analysis.

### 3.3. Hazard Assessment of Soil Heavy Metals

Table 2 shows that the mean contents of Zn, Hg, and Cd exceed soil background values, but they are still lower than the Grade II criteria, which mean that the three metal elements are mainly affected by anthropogenic sources. The mean value of soil Zn 69.96 mg/kg was higher than its background value 62.0 mg/kg but it did not exceed the limiting content 300 mg/kg of SEPA. The average value of Cd 0.15 mg/kg was higher than its background value 0.075 mg/kg. Phosphate fertilizers have been well known as the major external source of soil Cd [26, 27]. Especially Hg is 3.49 times the background value and mainly originated from industry and traffic sources. Cu, Ni, Pb, Cr, and As concentrations are lower than or approximately equal to their corresponding background values, which indicates that these elements are dominated by natural sources and human activities.

The heavy metal concentrations in Zhengding agricultural soil are compared with the data reported from other areas in the world in Table 2 [6, 14, 17, 20]. The mean content of Cu and As is lower than that for other areas. The average values of Pb, Hg, and Cd are similar to those of Beijing and Tianjin, but lower than in other areas. The mean concentration of Cu, Pb, Cr, As, and Cd was lower than those reported by Hu, and within the range reported by Qiao.

The Chinese Environmental Quality Standard for soils (GB 15618-1995) [24] and the soil background values of Hebei were adopted to evaluate the pollution degree. The soil is mainly alkaline in the investigated area. Grade II criteria for soil quality are established to protect agricultural production and to maintain human health. Across the investigated area, a wide range of soil heavy metal concentrations have been measured.

The analyzed results indicate that all of the metal concentrations are below the Environmental Protection Administration for soil in China. The *P*_*i*_ of soil heavy metals and the Nemerow *P* values, range, median values, and CV in the 100 topsoil samples are shown in Table 3. On average, the *P*_*i*_ indices for Cu, Zn, Ni, Pb, Cr, Hg, As, and Cd were 0.212, 0.233, 0.417, 0.054, 0.231, 0.076, 0.246, and 0.246, respectively. Different heavy metal concentrations of single pollution index are in an order of Ni > Cd = As > Zn > Cr > Cu > Hg > Pb. The single pollution index showed that the Ni pollution intensity was strong. Cd, Cr, and Pb are considered as the most important environmental pollutants in agricultural soils because of the potential harmful effects they may have on food quality and health of soil.

The Nemerow integrated pollution index (*P*) in this area is 0.305, which is lower than 1, meaning that heavy metal exposure through the food chain does not have considerable consequence and is generally safe.

### 3.4. Semivariogram Analysis

**Figure 3:**
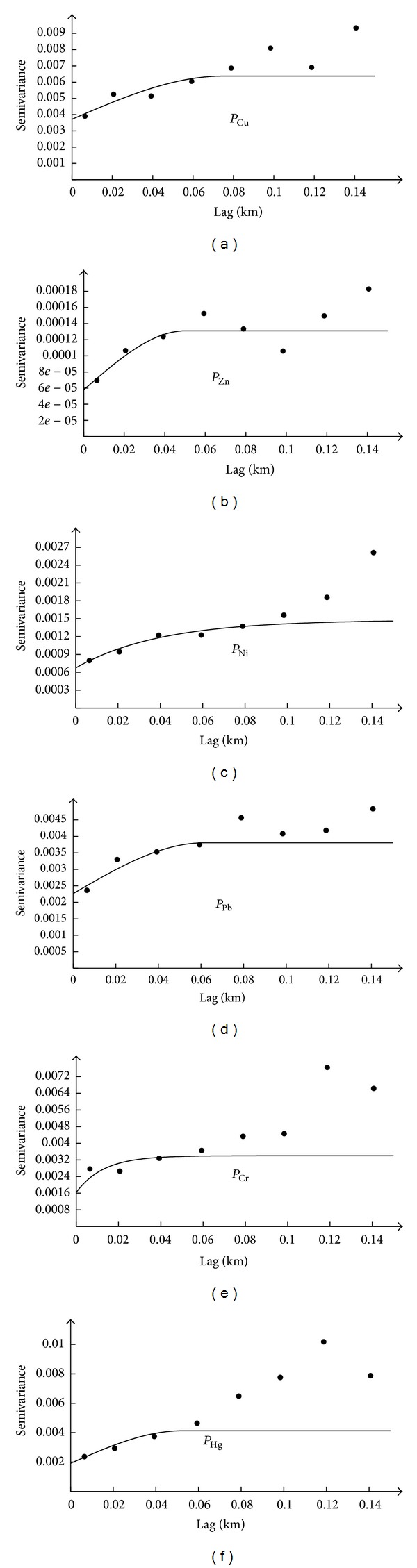
Semivariograms model of soil heavy metal single pollution index.

The semivariogram model of soil heavy metal single pollution index at both orientations is given in Figure 3. Theoretical models were then employed to interpret the experimental semivariograms and the model with the best fitting was chosen [22]. *P*_Ni_ and *P*_Cr_ were fitted with the exponential model, and the other four heavy metal single pollution indexes were all best fitted with the spherical model.

The attributes of the semivariograms for each soil heavy metal single pollution index were also summarized in Table 4. All of the Nug/Sill ratios were less than 59.74%, indicating random heterogeneity. The nugget effect may be caused by random factors such as data deviations, agricultural activities, or sample density. Nugget contributions highlighted the stronger spatial correlation in Table 4. The range of semivariograms for soil heavy metal single pollution index ranged from 0.0384 km to 0.1192 km. This confirmed the rational of the sampling density.

## 4. Conclusions

The single pollution index, integrated pollution index, and sources of the heavy metals Cu, Zn, Ni, Pb, Cr, Hg, As, and Cd in agricultural topsoil samples collected from Zhengding have been investigated in this work. The mean values of single pollution index and integrated pollution index are less than 1 in the area.

The mean values of Zn, Ni, Pb, Cr, Hg, As, and Cd in the analysed soils do not exceed the limited second grade criteria environmental quality standard for soils in China (GB 15618-1995), which means that the soil in this area is not polluted. Only Zn, Hg, and Cd present higher values in some cases. The mean values of soil Zn, Hg, and Cd were higher than the, respectively, background values. Agrochemical inputs may play the most important role for the input of Zn and Cd. The risk of Hg and Cd accumulation requires further attention and monitoring.

Multivariate statistics is found to be a powerful tool to identify the main factors determining the variability of geochemical data and interpret the measurement results. Variation of Cu, Zn, and Cd concentrations is controlled by anthropogenic intense agriculture activities. The concentrations of Pb and As in agricultural soil are abnormalities mainly affected by aerial deposits from gasoline exhausts, while the concentrations of Ni and Hg in agricultural soil are mainly affected by natural parent material and human activities.

## Figures and Tables

**Table:** (a) Total variance explained

Component	Initial eigenvalues			Extraction sums of squared loadings			Rotation sums of squared loadings		
	Total	% of variance	Cumulative %	Total	% of variance	Cumulative %	Total	% of variance	Cumulative %
1	2.700	33.752	33.752	2.700	33.752	33.752	2.187	27.338	27.338
2	1.464	18.304	52.056	1.464	18.304	52.056	1.639	20.484	47.822
3	1.264	15.797	67.853	1.264	15.797	67.853	1.602	20.030	67.851
4	0.893	11.160	79.013						
5	0.630	7.871	86.884						
6	0.479	5.987	92.871						
7	0.362	4.523	97.394						
8	0.208	2.600	100.000						

**Table:** (b) Component matrixes

Element	Component matrix			Rotated component matrix		
	PC1	PC2	PC3	PC1	PC2	PC3
Cu	0.838	0.231	0.117	**0.648**	0.585	0.086
Zn	0.804	0.236	−0.325	**0.875**	0.207	0.008
Ni	0.549	−0.593	0.335	0.125	0.367	**0.785**
Pb	0.317	0.028	0.727	−0.153	**0.756**	0.182
Cr	0.692	−0.355	−0.148	0.565	0.114	0.544
Hg	−0.041	0.850	0.101	0.085	0.319	**−0.791**
As	0.428	0.381	0.380	0.205	**0.637**	−0.156
Cd	0.536	0.096	−0.572	**0.771**	−0.166	0.013

**Table 2:** A summary of heavy metal concentrations in agricultural soil for various areas (mg/kg).

Area	Cu	Zn	Ni	Pb	Cr	Hg	As	Cd	References
Zhengding	21.22	69.96	25.04	18.80	57.77	0.08	6.16	0.15	This study
Background value	21.7	62.0	28.8	20.0	63.9	0.023	12.1	0.075	Yang et al. (2009) [18]
Grade II	100	300	60	350	250	1.0	25	0.6	SEPA (1995) [24]
Beijing	26.08	61.18	24.01	18.81	67.77	0.079	7.68	0.24	Hu et al. (2004)
Beijing, Tianjin	28.2	71.0	NA	18.7	52.3	0.092	7.9	0.145	Qiao et al. (2011) [15]
Alicante	22.5	52.8	20.9	22.8	26.5	NA	NA	0.34	Mic$\overset{´}{\text{o}}$ et al. (2006) [6]
Ebro	17.3	57.5	20.5	17.5	20.3	0.036	NA	0.42	Rodriguez et al. (2006)
Huizhou	21.82	66.15	20.52	65.38	43.01	0.24	12.76	0.12	Cai et al. (2012) [9]

Grade II (the value for protection of agricultural production and human health).

**Table 3:** Descriptive statistics of soil heavy metals pollution indices.

Variable	*P*_*i*_								*P*
	Cu	Zn	Ni	Pb	Cr	Hg	As	Cd	
Min.	0.111	0.154	0.215	0.035	0.131	0.018	0.087	0.15	0.091
Max.	0.332	0.293	0.608	0.117	0.354	0.374	0.396	0.483	0.521
Mean	0.212	0.233	0.417	0.054	0.231	0.076	0.246	0.246	0.305
Kurt.	1.403	0.430	−0.283	9.317	1.959	9.669	0.177	3.142	1.968
Skew.	0.352	0.073	−0.185	2.108	0.762	2.769	−0.116	1.476	0.086
St.d	0.034	0.024	0.077	0.011	0.035	0.060	0.060	0.067	0.121
C.V.	16.04	10.30	18.47	20.37	15.15	78.95	24.39	27.24	39.67

St.d: standard deviation, C.V.: coefficient variation.

**Table 4:** Spatial correlation for heavy metals.

	Model	Nugget *C*_0_	Sill *C* + *C*_0_	*C*_0_/(*C* + *C*_0_) (%)	Range (km)
*P*_Cu_	Spherical	0.0037	0.0064	58.40	0.0746
*P*_Zn_	Spherical	5.8*e* − 05	0.0001	44.25	0.05
*P*_Ni_	Exponential	0.0007	0.0015	45.58	0.1192
*P*_Pb_	Spherical	0.0023	0.0038	59.74	0.0613
*P*_Cr_	Exponential	0.0016	0.0034	48.24	0.0384
*P*_Hg_	Spherical	0.0019	0.0041	46.49	0.0521
